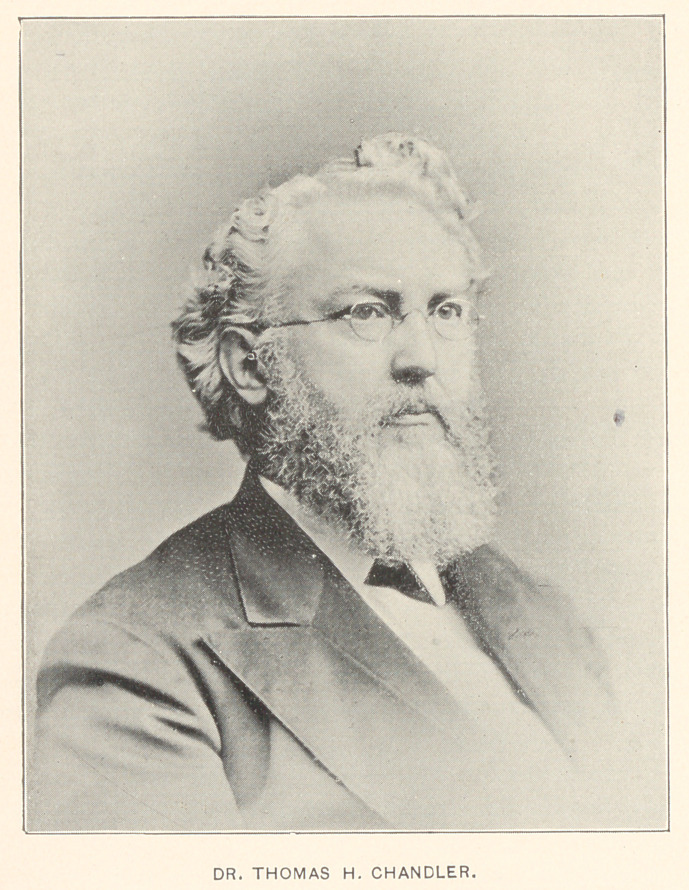# Resolutions of Respect to the Memory of Dr. Thomas H. Chandler

**Published:** 1896-01

**Authors:** 


### RESOLUTIONS OF RESPECT TO THE MEMORY OF DR. THOMAS H. CHANDLER.

In the death of Thomas H. Chandler, A.M., D.M.D., the Harvard Odontological Society recognizes the loss to the dental profession of a man of rare attainments, and to the city of Boston of one of her most honored and respected citizens.

His early education was received in the public schools of this city, and upon the conclusion of his preparatory studies in the Boston Latin School, he entered Harvard College, whence he graduated as a Phi Beta Kappa man in the class of 1848.

As a mark of the esteem in which he was held by his classmates in college he was elected to the presidency of the Hasty Pudding Club and to the class secretaryship,—a life position.

His connection with the Harvard Dental School commenced upon its institution, in 1869, when he received the appointment of adjunct professor of mechanical dentistry; and upon the resignation of Dr. N. C. Keep, in 1872, he was promoted to a full professorship, with the degree of D.M.D., *honoris causa.*

Upon the death of Dr. Thomas B. Hitchcock, in 1874, Dr. Chandler was elected to fill the vacant deanship, a position which he held up to the time of his decease, and the duties of which, for twenty-one years, were discharged with great interest in and fidelity to the school.

Dr. Chandler was a man who was universally beloved and respected by both students and his associates upon the Faculty, and by all who came into professional contact with him.

His own great love and thirst for knowledge served only as a stimulus for imparting it to others, and in the truest and best sense of the word it can be said of him that he was pre-eminently a *teacher.*

The Harvard Odontological Society, bearing in mind therefore with grateful appreciation his long years of untiring devotion and service to the interests of the school, and the imprint upon the calling of dentistry which such a life as his must always leave behind it, desires to place upon record the following resolution:

*Resolved*, that in the death of Dr. Chandler, the members of this Society recognize individually that they have lost not only an esteemed brother dentist, but a warm personal friend.

That to his family we extend our most sincere and heart-felt sympathy.

That a page of our records be set aside in honor and affection to his memory.

EDWIN C. BLAISDELL,
CHARLES H. TAFT,
WILLIAM H. POTTER,
*Committee.*

BOSTON, November 21, 1895.

## Figures and Tables

**Figure f1:**